# Analysis of somatic mutations across the kinome reveals loss-of-function mutations in multiple cancer types

**DOI:** 10.1038/s41598-017-06366-x

**Published:** 2017-07-25

**Authors:** Runjun D. Kumar, Ron Bose

**Affiliations:** 1 Division of Oncology, Department of Medicine, Washington University School of Medicine, 660S Euclid Ave, St. Louis, MO 63110 USA; 2 Computational and Systems Biology Program, Washington University in St. Louis, St. Louis, USA

## Abstract

In this study we use somatic cancer mutations to identify important functional residues within sets of related genes. We focus on protein kinases, a superfamily of phosphotransferases that share homologous sequences and structural motifs and have many connections to cancer. We develop several statistical tests for identifying Significantly Mutated Positions (SMPs), which are positions in an alignment with mutations that show signs of selection. We apply our methods to 21,917 mutations that map to the alignment of human kinases and identify 23 SMPs. SMPs occur throughout the alignment, with many in the important A-loop region, and others spread between the N and C lobes of the kinase domain. Since mutations are pooled across the superfamily, these positions may be important to many protein kinases. We select eleven mutations from these positions for functional validation. All eleven mutations cause a reduction or loss of function in the affected kinase. The tested mutations are from four genes, including two tumor suppressors (TGFBR1 and CHEK2) and two oncogenes (KDR and ERBB2). They also represent multiple cancer types, and include both recurrent and non-recurrent events. Many of these mutations warrant further investigation as potential cancer drivers.

## Introduction

Paired tumor-normal exome sequencing has revealed millions of somatic mutations across many thousands of patients^1^. Of these mutations, it is likely that only a small minority have a biological impact, while the majority of mutations are incidental to cancer development^2^. Identifying mutations that impact tumor biology and using this knowledge to guide experiments or therapeutic decision-making is a major goal.

Although the specific biologic effects of many mutations are unknown, many strategies rely on aggregating mutations to draw biological conclusions. For instance, mutations can be drawn from several genes to identify gene networks and pathways that are related to tumor growth^3^. Many tools also query mutations at the gene level to identify genes with non-random patterns of mutations that are likely related to cancer development^4, 5^. As the number of mutations increases, even regions within proteins can be assessed^6^, and clustered mutations can be detected^7^. Even though knowledge of specific mutations may be lacking, these approaches can guide researchers towards the most promising subsets of mutations for further study. However, one limitation of these approaches is that they operate genome-wide, often without taking into account relevant knowledge of specific gene families or protein types.

One particularly well-studied gene superfamily is protein kinases. These are a set of evolutationarily conserved phosphotransferases. There are approximately 500 protein kinase domains encoded in the human genome, spread between roughly 485 genes. These signaling molecules have well-known links to a variety of human diseases, and particularly to cancer due to their widespread functions in regulating cell behaviors^8, 9^. Several strategies for identifying biologically active mutations in protein kinases have been developed by focusing on characteristics specific to kinases^10^.

Torkamani and Schork observed that known disease-causing mutations are not randomly distributed throughout these proteins and developed a machine-learning method for identifying these mutations^11–13^. When applied to cancer mutations, they observed that predicted functional mutations clustered in hotspots, suggesting that functional mutations may be shared among protein kinases^14^. Recent studies continue to use machine-learning and kinase-specific data to improve the identification of functional mutations in kinases^15, 16^. KinView is a more recent method that allows mutations to be mapped across alignments and incorporates additional annotations. It is an interactive visualization program that was used to identify a loss-of-function mutation in PKCβ, a kinase which functions as a tumor suppressor gene^17^.

Another approach is to seek common effects of functional mutations. Dixit and colleagues demonstrated over several studies that activating protein kinase mutations shift the active-inactive equilibrium towards the active conformation, and that this is broadly true in many kinases^18–20^. Furthermore, they identified the catalytic and activation loops as particularly prone to gain-of-function events^21, 22^. Analogously, Olow *et al*. showed that nearly half of phosphorylation sites in the kinome-reactome are somatically mutated in at least some cancers^23^. This suggests that mutations with functional consequences may affect kinase substrates in addition to kinase enzymes.

It is clear that mutations occurring in one protein kinase can be used to draw inferences in another, and that biologically active protein kinase mutations may have some distinct characteristics which can be used to better identify them. However, these kinase-specific methods rely on prior structural knowledge, sets of labeled training mutations, or curated reaction datasets that limit generalizability beyond kinases.

In this study, we propose an alternative approach that relies only on unlabeled somatic mutations and an alignment of related genes or domains, which in principle is generalizable to other settings besides kinases. Rather than use prior knowledge of protein structure or post-translational modifications to find functional mutations, we first pursue the reverse task: using observed mutations and a protein kinase alignment to develop a functionality map of the human kinome. To do so, we design a series of statistical tests to identify aligned positions with non-random mutations, using our previous study of cancer genes as a starting point^5^. This strategy has not been used in prior studies of kinases or other gene families. We identify 23 homologous positions with non-random mutations, which is a novel finding in the field. We functionally assess eleven previously untested mutations across four genes by introduction into cell lines, and find that all eleven cause some reduction-of-function (ROF).

## Results

### Datasets

We used dGene to identify genes that have kinase domains, ultimately drawing 486 kinase domain sequences from 471 unique genes from Uniprot^24, 25^. These kinase domains were aligned using ClustalOmega with default settings^26^. The default settings are quite permissive to gaps in the alignment; this is acceptable for our purposes, since the analysis assumes that aligned residues have homologous functions, and a more stringent alignment may violate the assumption. To ensure the quality of the alignment, we compared it with results produced by alternate aligners including COBALT and MUSCLE, as well as older, manually curated alignments from kinase.com, and found that all were nearly identical^27–29^. We also manually examined the alignment to ensure major structural regions were aligned properly. The final alignment has 1808 positions (alignment available in Supplementary Table 1).

**Figure 1:**
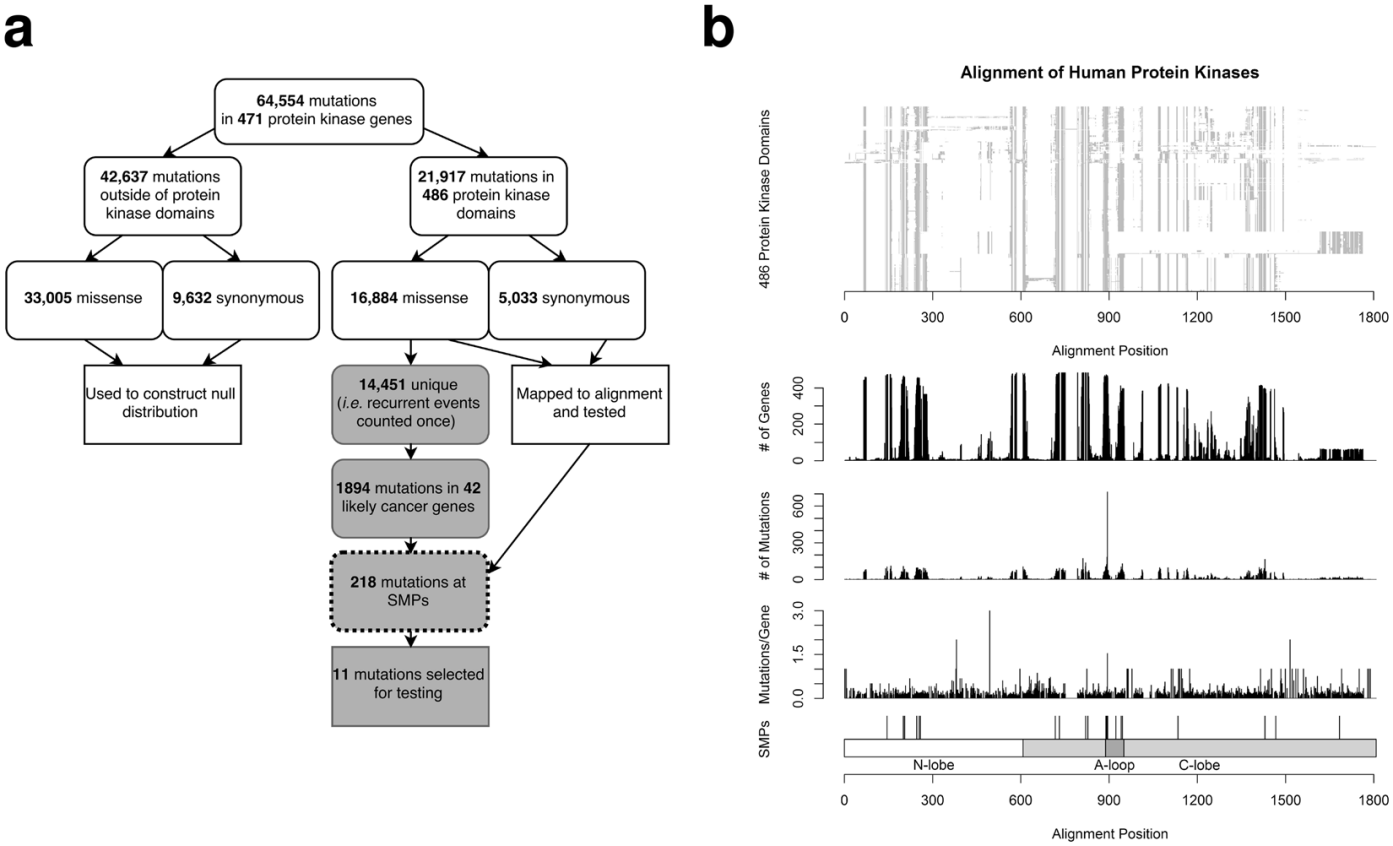
Study and data overview. (**a**) The use of mutations in the study. The process of choosing mutations for experimentation is in grey; the use of Significantly Mutated Positions (SMPs) is outlined. (**b**) Mapping of mutations to the protein kinase alignment. The location of 23 identified SMPs is indicated at the bottom, as well as the major regions of the aligned domains.

We draw 64,554 point mutations in these genes from our previous study, updated with additional mutations from the cBio portal (Fig. 1a, Supplementary Table 2)^5, 30^. 21,917 of the mutations map to the kinase domains, while the remainder are outside the kinase domain. Duplicate mutations from multiple sources were removed. We limit scope to just point mutations (missense and silent changes), because other types of mutations like insertions and deletions often cannot be mapped to a single position on the alignment. 14,665 silent mutations are included in all *in silico* analyses. Positions that are systematically enriched or depleted for silent mutations may be under negative or positive selection, respectively, making these events a valuable source of information^31, 32^. Moreover, there is evidence that some silent mutations have important functional consequences at the protein level^33, 34^. The mutations of our dataset come from 8,674 distinct patients, although the number of patients exome sequenced to generate these mutations is likely 10–20% higher, since some patients will have no mutations in any protein kinase.

### Testing Aligned Positions

Mutations were mapped onto the alignment of human kinase domains (Fig. 1b, Supplementary Table 2). Mutations in these genes which are outside the kinase domain are used to define the null distributions of test statistics, since they are produced by the same mutational processes as kinase domain mutations, but are unaligned. We developed a series of seven statistical tests to identify homologous positions with non-random mutation patterns, which can be calculated using basic approaches outlined in the Methods section. Importantly, these methods do not make assumptions regarding the neutrality of mutations used for the null distribution. The tests compare mutations at a given aligned position to unaligned mutations from outside the kinase domain; the goal is to identify aligned positions with mutations that appear non-random in relation to unaligned mutations. The tests include:

- *Mutation Number* – detects elevated numbers of mutations at an aligned position using a poisson distribution, given the observed mutation rates for residues aligned to the position.
- *Patients* – uses a chi-square statistic to detect deviations from expected patient distribution, given the number of mutations observed at the position.
- *Cancer Types* – uses a chi-square statistic to detect deviations from expected cancer type distribution, given the number of mutations observed at the position.
- *Reference Residues* – uses a chi-square statistic to detect deviations from expected distribution of mutated residues, given the observed residue substitution frequencies, residues aligned to the position, and the total number of observed mutations.
- *Variant Residues* – uses a chi-square statistic to detect deviations from expected distribution of variant residues, given the observed residue substitution frequencies, reference residues that are mutated, and the total number of observed mutations.
- *Cancer Genes* – detects sets of mutated genes that are enriched in predicted cancer genes, given the observed residue subsitution frequences, residues aligned to the position, and number of observed mutations.
- *Gene Relatedness* – detects sets of mutated genes that are more closely related than expected, given the observed residue substitution frequences, residues aligned to the position, and number of observed mutations.

### Constructing a Functionality Map

Since the tests require multiple mutations and genes to be calculated, they were applied to the 831 positions (of 1808 total) that had mutations in at least two genes. The p-values from the tests were then combined using the Fisher procedure to produce a single p-value for the position^35^. The Fisher procedure (see methods for details) is commonly used to combine p-values in the context of meta-analyses^36^, but has also been used to produce consensus scores from multiple tests^35^. These Fisher p-values were then adjusted for multiple-testing to control the false discovery rate (FDR)^37^. We found 23 significantly mutated positions (SMP) with FDRs less than 0.10 (Table 1, Supplementary Table 3, Supplementary Table 4).

**Table 1:** Significantly Mutated Positions. The 23 significantly mutated positions are listed, as well as their aligned position, kinase region and final Fisher FDR. The corresponding residues from several well-known protein kinases, as well as kinases used in this study are also indicated. Kinase regions β3-αC and αC-β4 refer to the β3-αC loop and αC-β4 loop, respectively. Positions bearing well-known functional mutations are underlined; positions corresponding to mutations to be tested *in vitro* are bolded and italicized. The complete listing of aligned positions and corresponding positions for all genes are in Supplementary Table 4, and additional summary statistics for the 23 SMPs are in Supplementary Table 5.

SMP	Aligned Column	Kinase Region	Fisher FDR	ALK	BRAF	EGFR	FLT3	TGFBR1	CHEK2	KDR	ERBB2
1	145	P-loop	2.47E-02	G1128	G469	G724	G622	G217	G232	G846	G732
2	200	β3-αC	6.22E-02	A1148	A481	A743	A642	A230	A247	A866	A751
3	205	β3-αC	4.87E-04	L1152	L485	L747	L646	F234	I251	L870	L755
4	246	αC-helix	5.87E-02	D1163	A497	E758	A657	***S241***	N269	A881	E766
5	254	αC-helix	5.12E-02	I1171	L505	M766	M665	Y249	L277	L889	M774
6	258	αC-β4	2.68E-02	F1174	—	V769	L668	V252	L280	I892	V777
7	717	C-lobe	2.68E-02	—	—	—	L780	—	—	K997	—
8	731	C-lobe	6.55E-02	A1230	A557	C818	A792	A306	F328	S1009	C826
9	820	C-loop	5.30E-03	R1253	N580	R841	R815	K337	E351	***R1032***	R849
10	828	C-loop	5.87E-02	L1256	F583	L844	L818	L340	L354	L1035	L852
11	889	A-loop	3.79E-05	F1271	F595	F856	F830	L352	F369	F1047	F864
12	891	A-loop	9.46E-09	M1273	L597	L858	L832	***L354***	H371	L1049	L866
13	892	A-loop	6.61E-03	A1274	A598	A859	A833	A355	***S372***	A1050	A867
14	893	A-loop	2.83E-09	R1275	T599	K860	R834	V356	***K373***	R1051	***R868***
15	894	A-loop	2.83E-09	D1276	V600	L861	D835	R357	I374	D1052	L869
16	895	A-loop	6.39E-02	I1277	K601	L862	I836	H358	L375	I1053	L870
17	923	A-loop	9.42E-05	K1285	S605	A871	V844	—	—	R1061	A879
18	941	A-loop	2.47E-02	V1293	I617	I878	V852	K376	P388	L1069	I886
19	945	A-loop	8.13E-08	P1297	A621	A882	A856	A380	**A392**	A1073	A890
20	1134	C-lobe	2.47E-02	S1324	—	T909	S883	R413	—	***S1100***	T917
21	1430	C-lobe	5.12E-02	R1373	R704	R958	R933	R482	R474	R1150	***R966***
22	1467	C-lobe	2.68E-02	C1386	—	M971	—	—	—	—	M979
23	1683	C-lobe	2.68E-02	—	—	—	—	—	—	—	—

One possible shortcoming of the Fisher procedure is that it may prioritize positions with one extremely small p-value over others with multiple borderline p-values^36^. Therefore, we scrutinized the results to determine the contribution of each test to detecting SMPs (Supplementary Figure 1). If we consider a p-value of less than 0.05 a positive result, *Mutation Number* detected the most SMPs (20 SMPs detected of 23 total; Supplementary Figure 1A). However, *Cancer Genes*, *Gene Relatedness*, *Cancer Types* and *Patients* all detected more than 5 SMPs each. *Variant* and *Reference Residues* contributed the least to detecting SMPs, with 4 SMPs detected by each. More importantly, we found that all but one SMPs were detected by multiple tests (Supplementary Figure 1B and C), and 11 of 23 were detected by three or more tests. In contrast, of 808 columns that were not identified as SMPs, only 37 were detected by two or more tests. Overall, it appears that most SMPs detected by the Fisher procedure have at least modest support from multiple tests.

### Characterizing SMPs

SMPs are exceptional positions and differ markedly from other positions in the alignment. The average SMP had 377 aligned domains, versus only 75 across the entire alignment (Supplementary Table 5). They also had more mutations (117 versus 12) and more mutated genes (61 versus 10) than the average aligned position. Overall, SMPs had about twice the average number of mutations per aligned domain (0.31 versus 0.16). This increased number of mutations reflects both a greater degree of recurrence (1.9 mutations per mutated gene at SMPs versus 1.25 elsewhere), as well as more genes that are mutated at SMPs (16% of aligned genes are mutated at SMPs, versus 13% elsewhere). SMPs were also slightly more conserved that most positions. SMPs had an average entropy score of 2.29, versus 1.37 for all 831 tested positions and 0.68 for all positions. However, entropy is markedly affected by the number of genes aligning at a given position. 18 SMPs had at least 350 aligned genes; the mean SMP entropy of these SMPs was 2.52, while 181 non-SMPs with over 350 aligned genes had entropy scores of 2.86 on average. However, these are only summary statistics, and many individual SMPs go against these trends (Supplementary Table 5).

**Figure 2:**
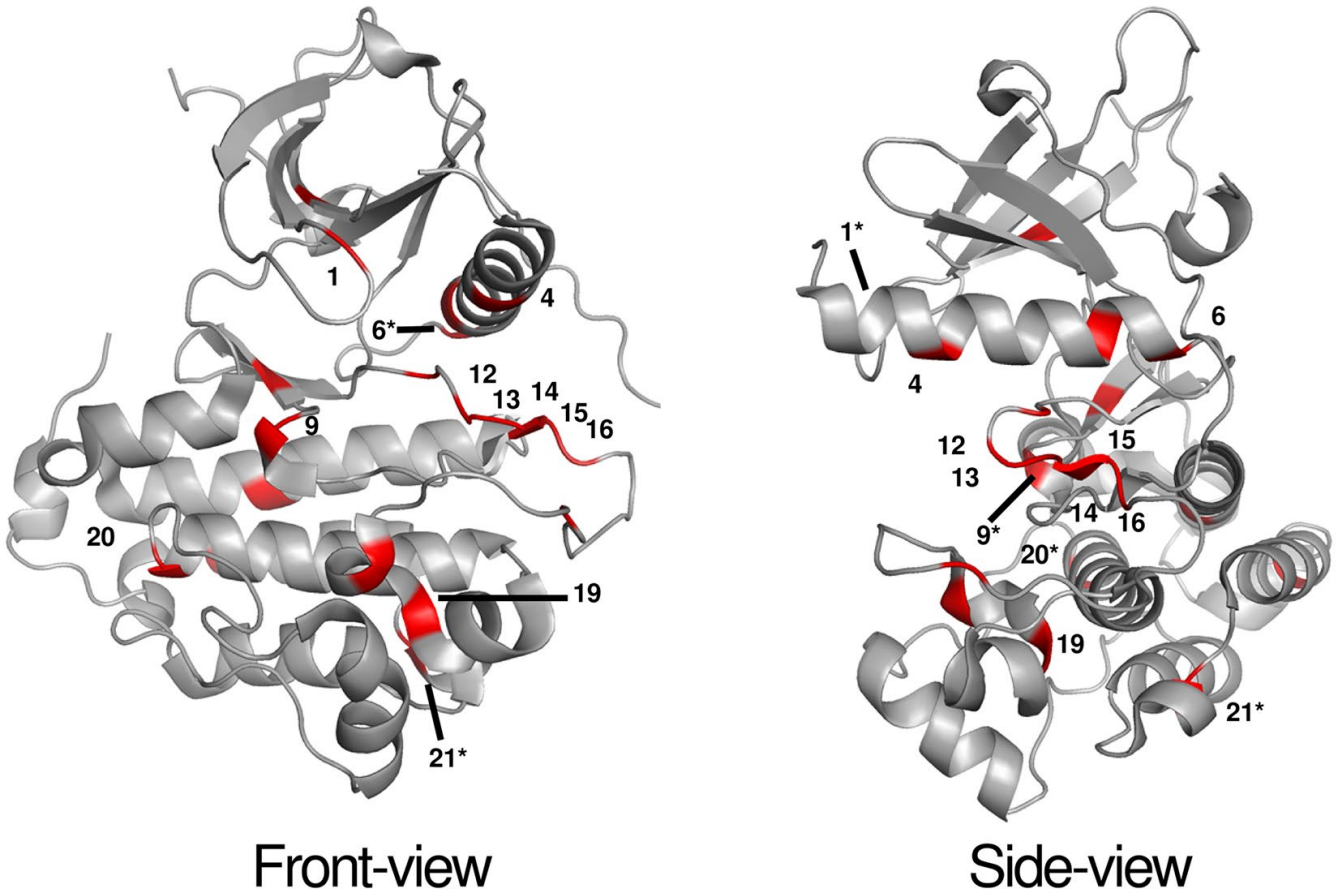
Significantly Mutated Positions as they appear on the EGFR kinase crystal structure. 20 of 23 SMPs which have corresponding positions on the EGFR kinase crystal structure are highlighted in red. Positions bearing *well-known functional mutations* and mutations to be validated *in vitro* are labeled following the numbering of Table 1 and include: 1) *BRAF G469A*/*V*. 4) TGFBR1 S241L. 6) *ERBB2 V777L*, *ALK F1174L/C/V/I*. 9) KDR R1032Q. 12) *EGFR L858R*, TGFRB1 L354P. 13) CHEK2 S372F/Y. 14) *ALK R1275Q*, ERBB2 R868W, CHEK2 K373E. 15) *BRAF V600E*, *EGFR L861Q*/*R*, *FLT3 D835Y*/*V*/*H*/*E*. 16) *BRAF K601E*, *FLT3 I836M*/*H*. 19) CHEK2 A392S/V. 20) KDR S1100F. 21) ERBB2 R966C. *Position is partially or fully obscured.

When viewed against the known structure of kinase domains, these SMPs compose a map of regions that may be important to kinase function. In Fig. 2, we project these positions onto the EGFR kinase domain crystal structure. One notable group are SMPs 11–19 in Table 1 and Fig. 2; these are all very well-known activation loop (A-loop) residues, and many are known to host important functional mutations^38^. Additionally, SMP 1 (aligned position 145) is located in the nucleotide binding P-loop, SMPs 4 and 5 (aligned positions 246 and 254) are in the αC-helix, SMPs 2, 3 and 6 (aligned positions 200, 205, and 258) are in the loops either N- or C-terminal to the αC-helix (the β3-αC loop and the αC-β4 loop, respectively), and SMPs 9 and 10 (aligned positions 820 and 828) are located in or adjacent to the catalytic loop (C-loop). Well-known functional mutations at each of these positions are listed in the legend for Fig. 2 (see underlined mutations) and a recent study by Foster *et al*. demonstrated how deletion mutations in the β3-αC loop (corresponding to SMPs 2 and 3) are able to activate BRAF, EGFR, and ERBB2 kinases^39^.

### Selecting Mutations for Validation

We first narrowed focus to just 14,541 unique missense mutations in the kinase domains (Fig. 1a). We further focus on the 42 protein kinases which we previously confirmed or predicted as cancer genes, reducing the candidates to 1894 mutations (genes had to have greater than even chance of being either an oncogene or tumor suppressor according to our previous study)^5^. Finally, we limited scope to the 23 SMPs, resulting in 218 candidate mutations.

We selected ten of these mutations for functional testing in cell culture (Table 2). We sought a mix of recurrent and non-recurrent events, mutations from diverse areas of the kinase domain, and a variety of cancer types. In particular, we tried to test mutations at several SMPs, and avoid mutations that were closely related to well studied functional mutations. Therefore, the mutations we selected represent a variety of novel hypotheses suggested by the functionality map. The mutations we selected include events in TGFBR1, CHEK2 and KDR, as well as the ERBB2 R868W mutation (Table 2). Five are non-recurrent, and seven are not homologuous to known functional mutations to our knowledge.

**Table 2:** Tested mutations. Key: strongly inactivating (↓↓↓), moderately inactivating (↓↓), modestly inactivating (↓). ^†^ERBB2 R966C was not directly observed in the dataset, but this amino acid substitution is common at this SMP in other genes (see text).

Gene	Mutation	Occurrences	Region	Effect on activity	SMP/Aligned Column
TGFBR1	S241L	5	αC-helix	↓↓↓	4/246
TGFBR1	L354P	1	A-loop	↓↓↓	12/891
CHEK2	S372F	1	A-loop	↓↓↓	13/892
CHEK2	S372Y	1	A-loop	↓↓↓	13/892
CHEK2	K373E	48	A-loop	↓↓	14/893
CHEK2	A392S	1	A-loop	↓	19/945
CHEK2	A392V	2	A-loop	↓↓↓	19/945
KDR	R1032Q	6	C-loop	↓↓↓	9/820
KDR	S1100F	7	C-lobe	↓↓↓	20/1134
ERBB2	R868W	1	A-loop	↓↓↓	14/893
ERBB2	R966C	0^†^	C-lobe	↓↓↓	21/1430

Our group specializes in ERBB2/HER2, and we have particular interest in mutations occuring in the terminal portion of the C-lobe. Since none of the mutations observed in this region occurred at an SMP, we identified additional mutations that otherwise did not meet the selection criteria. SMP 21 (position 1430 of the alignment) is one of the most downstream SMPs; although no mutation was observed in ERBB2 at this position, an R to C change occurred at this position 33 times in 23 different genes, including one observation of EGFR R958C. We therefore constructed ERBB2 R966C, which corresponds to this position.

Our chosen mutations also represent a variety of cancer types. They occur in a total of 73 patients with more than eleven distinct cancers (Supplementary Table 6). The CHEK2 K373E variant was split among many cancer types, but 17 patients with lung adenocarcinoma carried it. The KDR variants R1032Q and S1100F were predominantly observed in 11 melanoma patients. Finally, the TGFBR1 S241L and ERBB2 R868W mutations were found in colorectal patients.

### Experimental Results

Using a previously described retroviral transduction system^40^, we produced NIH 3T3 cells stably overexpressing both mutant and wild-type proteins for each of TGFBR1, KDR and ERBB2. We found that we could not stably overexpress wild type CHEK2 in this setting: cells retained the selection marker, but stopped expressing the construct. Instead, CHEK2 experiments were performed using transient transfection in HEK293T cells. TGFBR1, CHEK2 and KDR constructs were tagged with FLAG. All experiments were performed in duplicate or triplicate.

#### TGFBR1

**Figure 3:**
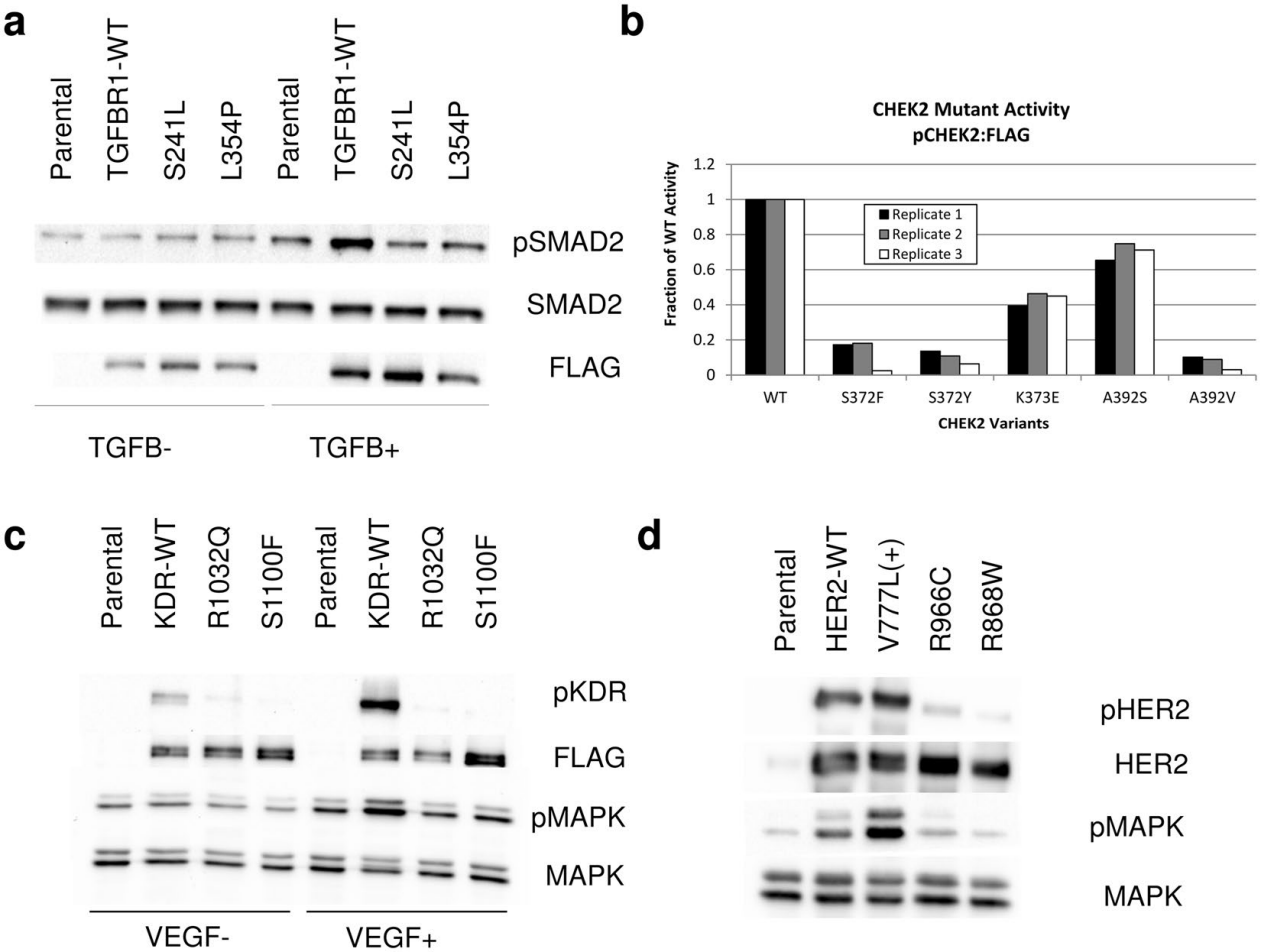
Functional validation of mutations in TGFBR1, CHEK2, KDR/VEGFR2, and ERBB2/HER2. (**a**) The mutations TGFBR1 S241L and L354P were tested in NIH 3T3 cells in the absence and presence of ligand. (**b**) The mutations CHEK S372F/Y, K373E, and A392S/V were tested by transient transfection of HEK 293 T cells. (**c**) The mutations KDR R1032Q and S1100F were tested in NIH 3T3 cells in the absence and presence of ligand. (**d**) The mutations ERBB2 R868W and R966C were tested in NIH 3T3 cells. Western blots have been cropped for visualization. See methods for details.

TGFBR1 (Transforming Growth Factor Beta Receptor 1) is a receptor S/T kinase. It has well appreciated functions in immune regulation as well as tissue remodeling. It is generally thought of as a tumor suppressor and acts to arrest the cell cycle^41^, although it can also act as a pro-tumor factor in later disease progression, particularly by causing increased cell invasiveness, proliferation and migration^42, 43^. We tested two mutations in this gene. We found that NIH 3T3 cells overexpressing TGFBR1 S241L and L354P had reduced signaling when exposed to the ligand TGFβ when compared with wild type (Fig. 3a).

#### CHEK2

Checkpoint 2 is a cytoplasmic S/T kinase that has important functions in cell cycle control, specifically in DNA damage and repair, and is a well appreciated tumor suppressor^44^. We transiently transfected HEK 293T cells with wild type CHEK2 and five variants. We confirmed previous observations that wild type CHEK2 is constitutively activated under these conditions, as judged by phosphorylation at the autophosphorylation site S516^45^. We found that CHEK2 S372F, S372Y, and A392V all had less than 15% of the wild type phosphorylation. The highly recurrent mutant K373E had 45% of wild type phosphorylation, while A392S had 70% (Fig. 3b; representative raw image Supplementary Figure 2).

#### KDR/VEGFR2

KDR/VEGFR2 (Vascular Endothelial Growth Factor Receptor-2) is a receptor tyrosine kinase (RTK). KDR is a well-established oncogene with crucial roles in angiogenesis, although there is evidence of an autocrine function as well^46^. We tested two mutations in this gene. We found that both the R1032Q and S1100F mutations markedly reduced function, as judged by levels of phospho-KDR and phospho-MAPK after exposure to the ligand VEGF (Fig. 3c).

#### ERBB2/HER2

ERBB2/HER2 is a member of the EGFR family of RTKs and a well known oncogene. Our lab has shown that point mutations in the HER2 kinase domain can trigger increased signaling and cell transformation in both breast^40^ and colorectal cell lines^47^. We found that HER2 R966C and R868W caused a reduction-of-function as judged by levels of phospho-HER2 and MAPK signaling (Fig. 3d).

### Analysis of Kinase Groups

Finally, using the classification scheme suggested by UniProt (Supplementary Table 7), we used the same procedures to identify additional SMPs within groups of related kinases (Supplementary Table 8). We found that groups with few members and mutations produced results that were highly sensitive to even single mutations; for this reason we limit the analysis to groups with more than 20 members and 2000 kinase domain mutations, excluding the atypical and “other” kinases, since they are highly heterogeneous.

The largest groups (over 50 members each) yielded relatively few group-specific SMPs. Among the five largest groups (AGC, CAMK, CMGC, STE, TYR), only 14 SMPs could be detected, all but two of which were identified in the main analysis. In the STE group (which includes MAP kinases), column 269 was identified; this position contains numerous recurrent mutations in the group, including P124S in MAP2K1 which is common in melanomas. The other group-specific SMP from these groups was column 951 in the CAMK group (which includes CHEK2). In contrast, the smaller tyrosine kinase-like group (TKL, 33 members, including TGFBR1 and BRAF) had 13 SMPs identified, 9 of which were not identified in the main analyses. These positions included columns 887, 888 and 890, corresponding to the N-terminal portion of the A-loop. These results suggest that there may be additional SMPs present in smaller kinase groups, but that additional data will be required to identify them reliably.

## Methods

### Statistical Tests

We developed a panel of statistical tests which can be used to identify non-random sets of mutations that occur at homologous positions in human kinases. Several of these tests are adapted from our previous study^5^. In many cases, null distributions are defined empirically (via permutation). Where needed, amino-acid substitution frequencies are defined by mutations that are outside kinase domains (but within genes bearing kinase domains) as these mutations are generated by the same mutational processes that produce the kinase domain mutations. Importantly, our method makes no assumptions regarding the functional status of these mutations; it merely assumes that mutations at some aligned positions will be enriched for functional events compared to unaligned mutations as a whole. That is, our method is tolerant to the fact that some non-kinase-domain mutations may be functional^48, 49^. This contrasts with prior methods which require presumably neutral mutations to define a null distribution, for instance by using silent mutations^7^. In some cases, the null distribution is also conditioned on the alignment and aspects of the observed mutations (for instance, most tests assume a fixed number of mutations).

Careful consideration was given to recurrent mutations which occur in more than one patient. These mutations are often presumed to have a functional effect^15^, but they may also be idiosyncratic to particular genes. Completely excluding recurrent mutations will likely remove many biologically important mutations from the dataset; but completely including them will likely make the analysis sensitive to positions with even a few recurrent mutations. Therefore, our panel includes tests that operate at three levels, which reflect different ways of handling recurrent events. Mutation-level tests (*Mutation Number, Patients, and Cancer Types*) include all mutations in the dataset, and consider recurrent events as non-redundant. Residue-level tests (*Reference Residues*, *Variant Residues*) treat identical amino-acid substitutions as redundant (e.g. CHEK2 K373E, which occurs 48 times in the dataset, is counted as a single event). Finally, gene-level tests (*Cancer Genes*, *Gene Relatedness*) treat mutations that occur at a single position in a gene as redundant (e.g. CHEK2 S372F and CHEK2 S372Y are treated as a single event). This approach should balance the value of recurrent mutations in identifying important positions against the risk of finding positions that are not broadly important to kinase function.

#### Mutation Number

In this simple test, we identify aligned positions with a higher-than-expected number of total mutations. All mutations are used, and the null is set using only non-kinase-domain mutations. We begin by defining the expected number of mutations per residue type (*r*) using the mutations and sequences that are outside of kinase domains:

1 $${E}_{r}=\frac{{O}_{r}}{{N}_{r}}$$

where *E*_*r*_ is the expected number of mutations per residue of type *r*, *O*_*r*_ is the observed number of mutations affecting residues of type *r* outside of the kinase domains, and *N*_*r*_ is the total number of residues of type *r* present in gene sequences, but outside of their respective kinase domains. Once the expectations per residue type are set, we calculate the expected number of mutations at each aligned position (*a*):

2 $${E}_{a}=\sum _{r}{E}_{r}{R}_{a,r}$$

where *E*_*a*_ is the expected number of mutations at an aligned position *a*; *E*_*r*_ is the expected number of mutations per residue type *r*, and *R*_*a,r*_ is the number of residues aligned at *a* of type *r*. We assume that the presence of mutations at each gene and aligned position can be modeled with a poisson distribution, parameterized by *E*_*r*_ for the appropriate residue type. It follows that the number of mutations for an entire aligned position is therefore also poisson distributed (since it is a sum of poisson variables), and parameterized by *E*_*a*_. By comparing the observed number of mutations at the position with the null distribution, we generate an upper tail p-value for the test.

#### Patients and Cancer Types

In these tests, we identify positions with mutations that are not randomly distributed among patients and cancer types, given the number of mutations observed at the position. They are calculated very similarly to one another, and are described in our previous study^5^. Both are calculated as chi-square goodness-of-fit tests, although both use empirical rather than theoretical distributions. Both tests use all mutations at the aligned positions. Unlike the other tests, the null distribution *includes mutations in kinase domains, as well as mutations outside kinase domains*.

Each mutation can be assigned to a patient (and cancer type), each of which has a certain mutation count associated with it (*c*). The mutation count is simply the number of times the patient (or cancer type) occurs in the dataset. Once each mutation has been associated with a value of *c*, we calculate the test statistic for each aligned position (*a*):

3 $${X}_{a}^{2}=\sum _{c}\frac{{({O}_{a,c}-{E}_{a,c})}^{2}}{{E}_{a,c}}$$

4 $${E}_{a,c}=\frac{{N}_{a}{N}_{c}}{N}$$

where *O*_*a,c*_ is the observed number of mutations at the aligned position from patients (cancer types) with mutation count *c*, *E*_*a,c*_ is the expected number of mutations at the aligned position from patients (cancer types) with mutation count *c*, *N*_*a*_ is the number of mutations at the position, *N*_*c*_ is the total number of mutations in the dataset from patients (cancer types) with mutation count *c*, and *N* is the total number of mutations in the dataset.

This statistic is compared to a null distribution, which is generated by calculating the statistic for random draws with replacement from the set of patient (cancer type) labels, holding the number of mutations fixed. The final output is an upper-tail p-value.

#### Reference Residues

This test identifies positions where mutated residues appear non-random. It is calculated as a chi-square goodness-of-fit test, but uses an empirical null distribution instead of a theoretical one. It is a residue-level test, and recurrent mutations with identical residue changes are removed. The null distribution is set with mutations from outside of kinase domains. We use the expected number of mutations per residue of each type (*E*_*r*_) that was used in *Number of Mutations*. We then calculate the test statistic for each aligned position (*a*):

5 $${X}_{a}^{2}=\sum _{r}\frac{{({O}_{a,r}-{E}_{a,r})}^{2}}{{E}_{a,r}}$$

6 $${E}_{a,r}={R}_{a,r}{E}_{r}$$

where *O*_*a,r*_ is the observed number of mutations at the aligned position from residues of type *r*, *E*_*a,r*_ is the expected number of mutations at the aligned position at residues of type *r*, and *R*_*a,r*_ is the number of residues at the aligned position *a* of type *r*.

This statistic is compared to a null distribution, which is generated by calculating the statistic for random draws with replacement from the set amino acid types (weighted by *E*_*a,r*_ for each residue type), holding the number of mutations fixed. The final output is an upper-tail p-value.

#### Variant Residues

This test is very similar to *Reference Residues*, but tests for positions where the newly produced amino acids appear non-random. It is calculated as a chi-square goodness-of-fit test, but uses an empirical null distribution instead of a theoretical one. It is a residue-level test, and recurrent mutations with identical residue changes are removed. The null distribution is set with mutations from outside of kinase domains. We then calculate the test statistic for each aligned position (*a*):

7 $${X}_{a}^{2}=\sum _{v}\frac{{({O}_{a,v}-{E}_{a,v})}^{2}}{{E}_{a,v}}$$

8 $${E}_{a,v}=\sum _{r}{P}_{r,v}{O}_{a,r}$$

where *v* is the type of variant residue and *r* is the type of reference residue. *P*_*r,v*_ refers to the probability that a mutation occurring at a residue of type *r* will result in a residue of type *v* (calculated based on the amino acid substitution frequencies observed outside of kinase domains), and *O*_*a,r*_ is the observed number of mutations at aligned position *a* with reference residues of type *r*.

This statistic is compared to a null distribution, which is generated by calculating the statistic for random draws with replacement of amino acid types (weighted by *E*_*a,v*_), holding the number of mutations fixed. The final output is an upper-tail p-value.

#### Cancer Genes

This test identifies positions with mutations that tend to occur in predicted cancer genes. It is a gene-level test, and multiple mutations that affect a single gene at a single position are only counted once. We associate each gene with a score that represents how likely the gene is to be related to cancer. Cancer genes have smaller scores on average (for details, see “UK Score” from our previous study^5^).

To perform the test, we calculate the average score for the genes that are mutated at a given aligned position. We generate a null distribution by calculating the average score for random draws of genes (weighted by the *E*_*r*_ that corresponds to each gene’s aligned residue at the given position). The result of the test is a lower-tail p-value.

#### Gene Relatedness

This test identifies positions where mutated genes have kinase domains that are more closely relate to one another on average than expected by chance, given the mutation patterns observed outside of kinase domains. It is a gene-level test, and mutations that affect a single gene at a given position are only counted once. The distance matrix of all kinase domains in the dataset was calculated from the phylogenetic tree produced by ClustalOmega when it produced the alignment.

To perform the test, we calculate the average pair-wise distance for all genes that are mutated at a given aligned position. We generate a null distribution by calculating the average pair-wise distance for random draws of genes (weighted by the *E*_*r*_ that corresponds to each gene’s aligned residue at the given position). The result of the test is a lower-tail p-value.

### Fisher Procedure

The Fisher procedure is used to combine the individual p-values into a single consensus score, as was done in OncodriveFM^35^. The statistic is calculated:

9 $${X}_{2k}^{2} \sim -2\sum _{i=1}^{k}\mathrm{ln}(pi)$$

where *k* is the number of tests being combined. The test statistic can then be used to generate an upper-tail p-value.

Unweighted methods like the Fisher procedure are often considered inferior to weighted methods like weighted Z-scores in meta-analytic problems^36^. However, it is important to note that there is no clear role for weighting in our problem, since we have no prior reason to regard one test as more reliable or powerful than any other, as they all rely on the same underlying dataset. Therefore an unweighted approach is most appropriate.

We did compare the Fisher method to unweighted Z-scores as discussed by Whitlock^36^. We found that the Fisher procedure and unweighted Z-scores produced highly correlated results (r = 0.903) at the 831 tested positions, and that a large majority of SMPs would be identified by either method. The Z-score method generally detected fewer positions at a given cut-off. For instance, if an FDR cutoff of 0.2 were applied to Z-score based p-values, there would be 26 positive results, 21 of which are among the 23 SMPs identified by the Fisher method at the cutoff of 0.1. Based on these observations, the unweighted Z-score method and the Fisher method identify the same positions as most likely to be significantly mutated, although the absolute p-values may differ slightly.

### Missingness and Data Handling

The only variable with notable missingness was Cancer Type, which ~20% of mutations lacked. We found that excluding these mutations from the *Cancer Types* test or including them under a “missing/other” category produced virtually identical results. The final analysis includes them as a separate category.

For genes with multiple isoforms, merging multiple datasets sometimes required mapping mutations to a common isoform. To do so, we selected the isoform that conserved the greatest number of mutations. Less than 1% of kinase domain mutations were discarded in this process. The supplementary materials indicate when the mapped isoform differs from the UniProt canonical isoform. In the body of the next and figures, we refer to mutations according to the canonical isoform.

### Experimental Procedures and Reagents

Experiments were performed as previously described^40^. Briefly, cDNA for KDR, TGBFR1 and CHEK2 were purchased from Addgene. ERBB2 cDNA was a gift from Dr. Dan Leahy (Johns Hopkins University, Baltimore). Mutations were introduced using QuikChange II site-directed mutagenesis (Agilent). Constructs were then shuttled into the pCFG5 retroviral vector (which includes a zeocin resistance marker and IRES-GFP sequence) using the In-Fusion HD cloning system kit (Clonetech), and verified by full-length Sanger sequencing. For KDR, TGFBR1 and CHEK2, a c-terminal FLAG tag was introduced. For ERBB2, TGFBR1 and KDR, retroviral particles were produced using ϕNX amphotrophic packaging cells. NIH 3T3 cells were spin-infected with virus, and selected under 10 μg/ml zeocin for 3 weeks. Fluorescence was confirmed at >95% by flow cytometry or >90% by microscopy. Cells were serum starved for 6 hrs before lysate harvesting for each of these three genes. Cells were treated or untreated with ligand prior to harvesting in the case of TGBFR1 (20 min induction, 5 ng/ml) and KDR (10 min induction, 10 ng/ml). In the case of CHEK2, transient transfections were performed using LTX and Plus reagent from Thermo Fisher, using the manufacturers standard protocol in HEK 293 T cells. Cells were lysed 24 hrs after transfection. Transfection efficiency was confirmed by microscopy as >50% in all cases.

ERBB2/HER2 signaling was assayed using pHER2 and pMAPK levels^40^. TGFBR1 activity was assayed using pSMAD2 levels^43, 50^. KDR activity was assayed using pKDR^51^ and pMAPK levels. CHEK2 was assayed with pS516, which is both an autophosphorylation site and necessary for full activation of CHEK2, and has been used previously as a proxy of CHEK2 activity^45, 52, 53^.

NIH 3T3 cells were acquired from the American Type Culture Collection (ATCC). HEK 293 T cells were a gift from Dr. Akhilesh Pandey (Johns Hopkins University, Baltimore). Antibodies used include HER2 from Thermo-Fisher (Ab-17), phospho-HER2 (pY1248) from Millipore (06–229), p44/42 MAPK from Cell Signaling Technologies (CST, 137F5), phospho p44/42 MAPK from CST (20G11), FLAG from Sigma-Aldrich (F3165), phospho-KDR (pY1175) from CST (19A10), phospho-SMAD2 (S465/467) from CST (138D4), SMAD2 from CST (D43B4), phospho-CHEK2 (pS516) from CST (#2669). Ligand included VEGF_165_ (#8065) from CST and TGFβ.

### Data Availability

All data generated or analysed during this study are included in this published article (and its Supplementary Information files).

## Discussion

In this study, we hypothesized that somatic cancer mutations could be used to identify important functional regions within proteins. Specifically, we focused on the superfamily of protein kinases, which are a conserved set of phosphotransferases that share homologous sequences and structural motifs. By mapping mutations onto the alignment of protein kinases and applying a panel of statistical tests, we were able to identify homologous positions that bear mutations which appear non-random. Since mutations are pooled across all superfamily members, these positions may be broadly important to the function of many different protein kinases.

We found 23 significantly mutated positions (SMPs) within the kinase alignment. SMPs were found throughout the kinase domains, with the strongest enrichment in the A-loop and other major positions located in and around the P-loop, the αC helix, and the catalytic loop. We tested eleven distinct mutations in several genes, including the oncogenes ERBB2 and VEGFR2 and the tumor suppressors CHEK2 and TGFBR1. We focused on highly novel mutations, including many that are rare or non-recurrent, and avoided mutations with that are closely related to well-studied functional mutations. All eleven mutations reduced signaling through the corresponding kinase. The mutations we tested were observed in 73 patients with eleven cancer types, with particularly large numbers of these mutations occurring in colorectal carcinomas, lung adenocarcinomas, and melanomas.

The fact that all eleven tested mutations reduced function is an important finding. It illustrates the importance of functional characterization of mutations, particularly given the diverse roles protein kinases play in cancer development^5^. In tumor suppressors, focus is often on deletions or truncations since loss-of-function events in tumor suppressors could act as tumor drivers. In this study, we found that both highly recurrent (CHEK2 K373E) and rare point mutations (CHEK2 S372F/Y and A392V, TGFBR1 S241L and L354P) in tumor suppressors can also cause loss- or reduction-of-function. Similarly, while it may be tempting to assume that recurrent point mutations in oncogenes are either neutral or gain-of-function, this work shows that these mutations can be loss-of-function (for instance, KDR R1032Q and S1100F). In contrast to tumor suppressors, loss-of-function events in oncogenes would seem to be poor candidates as tumor drivers. As it becomes more common for patients to have their tumors exome or genome sequenced, this knowledge will be crucial in identifying events that are most like to underpin their disease.

There are some important drawbacks to our approach. On a technical level, one limitation of this study is the focus on protein-level changes, which was necessary as DNA-level changes are not uniformly publicly available. However, our methods are in principle compatible with DNA-level data, and it would provide two major benefits. First, applying our framework to a DNA alignment and set of nucleotide changes would allow analysis of non-protein regions. Second, in protein-coding regions, the use of DNA-level changes would allow us to correct for codon structure, potentially improving the performance of our tests.

Another caveat to this analysis is that while it provides a precise location within a gene or sets of genes to search for functional events, it does not identify specific mutations for testing. We addressed this problem by manually selecting candidate mutations from SMPs for experimentation. However, numerous methods exist that provide complementary functionality and could be combined with the work of this study. For instance, several studies have focused on identifying “hotspot” regions of genes with high densities of mutations, sometimes taking protein structure into account^7, 54, 55^. These methods can be used to identify regions within specific genes for further study, but do not yet implicate specific residues. Functional impact predictors which use a variety of inputs to identify mutations that are likely to alter protein function have also been developed^56^, including by our own group^57^. However, impact predictors can have high rates of false-positive results, and are best used on limited sets of mutations with a high prevalence of functional events. Combining the methods developed in this study with other complementary approaches may provide an avenue for reliably identifying functional events in large genomic datasets.

There are other potential extensions to this study, encompassing multiple fields. We have tested only a small fraction of the mutations at the SMPs we identified. Direct follow up studies, particularly on ROF mutations in the tumor suppressors TGFBR1 and CHEK2 will be necessary before these mutations can be confirmed as *bona fide* cancer drivers. Many other mutations are found at other SMPs, and our results suggest that testing these mutations could be fruitful, particularly if present in genes with therapeutic implications. Our results also have implications for the structural understanding of kinase signaling: for instance, the ERBB2 R966C mutation demonstrates the importance of the C-lobe to kinase function, but the exact role this region plays is not fully understood.

Our methods can also be applied in other settings. Although we have focused on kinases, none of our methods are kinase-specific. Our analysis is equally compatible with other conserved gene or domain families of broad importance to cancer development, such as nuclear hormone receptors^58^ and G-protein coupled receptors^59^. Our methods will also become more precise as data volumes continue to increase. We found additional SMPs within specific groups like the TKL kinases, and more may exist in even smaller groups. New platforms that incorporate multi-sequence alignments with cancer mutation data will allow future analyses to be quickly iterated and focused on specific kinases^17^. Our methods can even be adapted to single genes, provided a sufficient density of observed variants.

In conclusion, we have demonstrated the use of somatic mutations to identify functional positions and mutations within gene families. We developed several statistical approaches for identifying positions with non-random mutations, aggregating mutations across homologous positions in the human kinome to do so. We identified 23 significantly mutated positions, and tested eleven mutations found at these positions from several genes. We confirmed all eleven as causing reductions in kinase function. Mutations that reduce the function of tumor suppressors are particularly promising as candidate cancer drivers, though other mutations at these SMPs warrant study as well. Our methods are highly extensible, providing a framework for using somatic cancer data to identify functionally important regions in proteins, and eventually identifying mutations that are relevant to cancer development and growth.

## Electronic supplementary material

Supplementary Figures 1-2

Supplementary Table S1

Supplementary Table S2

Supplementary Table S4

Supplementary Table S3 and S5 to S8
