# Supplementary material for: Analysis of somatic mutations across the kinome reveals loss-of-function mutations in multiple cancer types
Source: Sci Rep. 2017 Jul 25;7:6418. doi: 10.1038/s41598-017-06366-x (PMC5527104; doi:10.1038/s41598-017-06366-x)
Supplement: Supplementary file 1 — Supplementary Figures 1-2 [file 41598_2017_6366_MOESM1_ESM.pdf]

Supplementary Figures

Analysis of somatic mutations across the kinome reveals loss-of-function mutations in multiple cancer types

Runjun D. Kumar[1,2] and Ron Bose[1,3]

[1]Division of Oncology, Department of Medicine, Washington University School of Medicine, 660 S Euclid Ave, St. Louis, MO, 63110

[2]Computational and Systems Biology Program, Washington University in St. Louis

[3]Correspondence to rbose@dom.wustl.edu

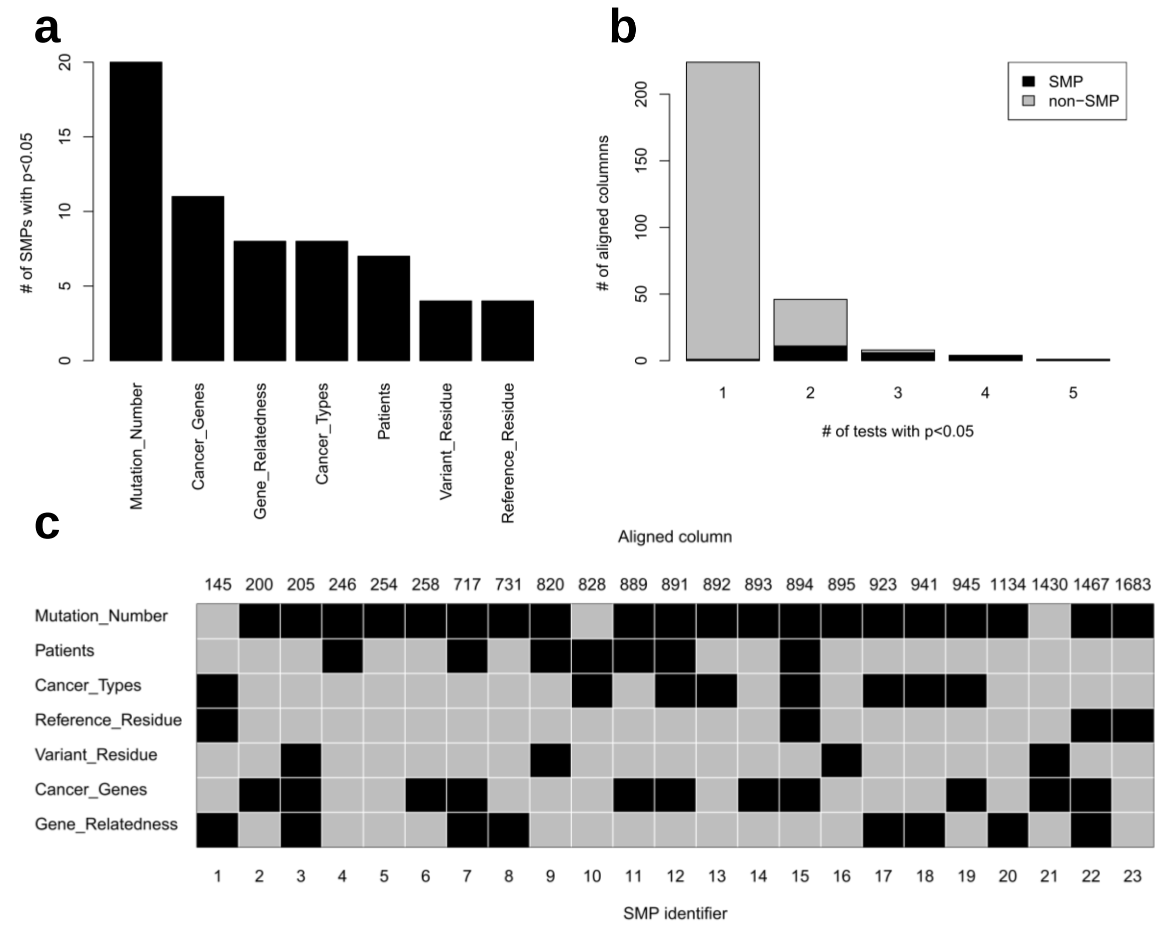

Supplementary Figure 1. Contribution of each test to detection of SMPs. A) Number of SMPs detected by each test at p<0.05. There are 23 SMPs total. B) Alignment columns detected by multiple tests at p<0.05 are often SMPs. C) For each combination of test and SMP, positive results are indicated (p<0.05). The SMP identifier represents numbering from Table 1.

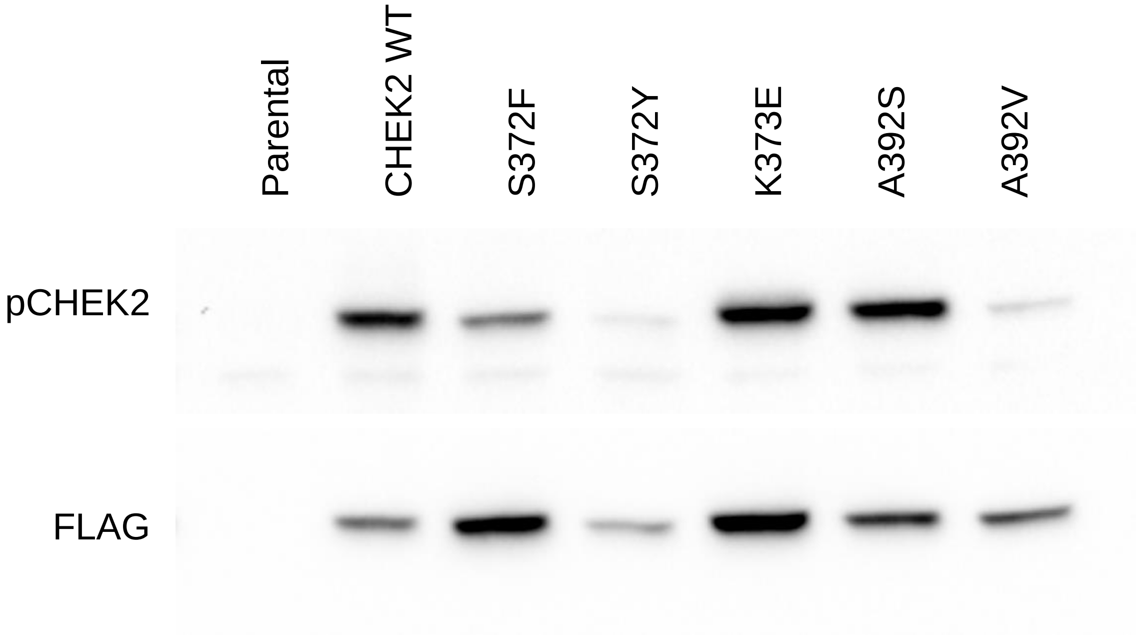

Supplementary Figure 2. CHEK2 activity in HEK 293T cells. CHEK-FLAG constructs were transiently transfected into cells and basal levels of phosphorylation assayed without induction. Image is representative of three replicates.
